# Prevalence and association of hepatitis C viremia in hemodialysis patients at a tertiary care hospital

**DOI:** 10.4103/0971-4065.59343

**Published:** 2009-10

**Authors:** S. K. Agarwal

**Affiliations:** Department of Nephrology, AIIMS, New Delhi, India

Sir,

I have read with interest the article by Jasuja *et al*., published in the recent issue of your esteemed journal.[1] I have some observation to make.

1. Indian references are conspicuously absent. Author has quoted references from France, Moldova and Syria and not from our own country. There is not a single reference from India, though there are 13 articles published in indexed journals alone.[2,14]
2. The author has stated in the introduction that duration of end-stage renal disease is one of the risk factors for prevalence of HCV in dialysis. I think this is not supported with the literature.
3. I fail to understand the basis of exclusion of patients with HCV who were receiving treatment. After all, they also create pool of patients contributing to overall prevalence.
4. Some centers are reusing dialyzer of HCV patients but in a different area. It is more important to specify that the reuse variable included in this study has taken this issue or not, and, if yes, then what was percentage of reuse being done in a separate area.
5. I would like to know the period of study; from the year of beginning to year of completion. 119 patients are from how many patients being dialysed during the period of study.
6. The hepatitis vaccination in hemodialysis patients is a 4-dose schedule. Hence, authors write that twice or more vaccination in 10 patients (8.4%) does not make any sense. Because it may still be inadequate vaccination. The author should mention full vaccination vs. inadequate vaccination. I am also not sure how HBV vaccination, which the author has analyzed, will affect HCV prevalence.
7. As the author performed the HCV test at the time of accepting patients on dialysis, they should mention the number of patients who already had HCV when they were accepted in their unit, and how many were actually new infection.
8. I am unable to understand how the author reached to the time for onset of CRF. In practical sense, we usually do not have serial values to determine the exact onset of CRF (CKD) in a majority of patients.
9. In the ROC curve, it is better to say at what duration of dialysis, patients will be HCV positive and with what probability. For example, after 20 months of dialysis, 50% chance is that a patient will be HCV positive. The findings presented by the authors seem odd. According to their criteria, additional dialysis from the 15^th^ to 16^th^ month, that is, for 1 month will change the prevalence of HCV from 7.4% to 45.2%. This, I think, is not possible. Authors themselves later conclude that increase in dialysis for one month increased the odds of HCV positivity by 1.06 times. This means that representation by the ROC criteria is not appropriate and can mislead the reader.
10. Authors have not mentioned other causes of increased liver enzyme, such as antitubercular drugs, while correlating the enzyme values with HCV positivity. About 12%-15% patients in any dialysis program in India have tuberculosis and they are on drugs. This issue has not been mentioned at all in the results or discussion.
11. In discussion, the authors are mentioning higher ALT as a risk factor for HCV infection, which is never a case. A higher ALT is in fact is the result of HCV infection.
12. The author describes that they do not isolate HCV patients and their HCV prevalence is 27.8%, which by any standard is not low. They should mention the new cases of HCV in their unit and only then they can explain that whether their not isolating HCV-positive patient is justified.

## References

[1] Jasuja S, Gupta AK, Choudhary R, Kher V, Agarwal DK, Misra A (2009). Prevalence and association of hepatitis C viremia in hemodialysis patients at a tertiary care hospital. Indian J Nephrol.

[2] Medhi S, Potukuchi SK, Polipalli SK, Swargiary SS, Deka P, Chaudhary A (2008). Diagnostic utility of hepatitis C virus core antigen in hemodialysis patients. Clin Biochem.

[3] Lakshmi V, Reddy AK, Dakshinamurty KV (2007). Evaluation of commercially available third-generation anti-hepatitis C virus enzyme-linked immunosorbent assay in patients on haemodialysis. Indian J Med Microbiol.

[4] Jindal N, Jindal M, Jilani N, Kar P (2006). Seroprevalence of hepatitis C virus (HCV) in health care workers of a tertiary care centre in New Delhi. Indian J Med Res.

[5] Reddy AK, Dakshinamurty KV, Lakshmi V (2006). Utility of HCV core antigen ELISA in the screening for hepatitis C virus infection in patients on hemodialysis. Indian J Med Microbiol.

[6] Chattopadhyay S, Rao S, Das BC, Singh NP, Kar P (2005). Prevalence of transfusion-transmitted virus infection in patients on maintenance hemodialysis from New Delhi, India. Hemodial Int.

[7] Reddy AK, Murthy KV, Lakshmi V (2005). Prevalence of HCV infection in patients on haemodialysis: Survey by antibody and core antigen detection. Indian J Med Microbiol.

[8] Reddy GA, Dakshinamurthy KV, Neelaprasad P, Gangadhar T, Lakshmi V (2005). Prevalence of HBV and HCV dual infection in patients on haemodialysis. Indian J Med Microbiol.

[9] Chandra M, Khaja MN, Hussain MM, Poduri CD, Farees N, Habeeb MA (2004). Prevalence of hepatitis B and hepatitis C viral infections in Indian patients with chronic renal failure. Intervirology.

[10] Saha D, Agarwal SK (2001). Hepatitis and HIV infection during haemodialysis. J Indian Med Assoc.

[11] Agarwal SK, Dash SC, Irshad M (1999). Hepatitis C virus infection during haemodialysis in India. J Assoc Physicians India.

[12] Gosavi MS, Shah SK, Shah SR, Pal RB, Saldanha JA, Banker DD (1997). Prevalence of hepatitis C virus (HCV) infection in Mumbai. Indian J Med Sci.

[13] Jaiswal SP, Chitnis DS, Naik G, Artwani KK, Pandit CS, Salgia P (1996). Prevalence of anti-HCV antibodies in central India. Indian J Med Res.

[14] Arankalle VA, Chadha MS, Jha J, Amrapurkar DN, Banerjee K (1995). Prevalence of anti-HCV antibodies in western India. Indian J Med Res.

